# Hybrid Spinal-General Anesthesia With a Supraglottic Airway in Severe Rheumatoid Arthritis: A Case Report

**DOI:** 10.7759/cureus.117042

**Published:** 2026-09-27

**Authors:** Shilpa Bajaj, Rahul Kalshan, Jitendra Shekhawat, Trisha Nidhi

**Affiliations:** 1 Anesthesiology, Holy Family Hospital, New Delhi, IND; 2 Anesthesiology, Safdarjung Hospital, New Delhi, IND

**Keywords:** case report, difficult airway, general anesthesia, i-gel, laparoscopic cholecystectomy, rheumatoid arthritis, spinal anesthesia, spontaneous ventilation, supraglottic airway

## Abstract

Rheumatoid arthritis (RA) can present significant challenges during airway management because of cervical spine involvement, restricted neck mobility, temporomandibular joint disease, and limited mouth opening. We report the perioperative anesthetic management of a patient with severe deforming RA and multiple predictors of an anticipated difficult airway undergoing laparoscopic cholecystectomy. A 54-year-old woman with long-standing RA had a Mallampati grade IV airway, approximately 1.5 cm mouth opening, a short neck, and markedly restricted cervical spine movement. A comprehensive difficult airway strategy was formulated with immediate availability of videolaryngoscopy, fiberoptic bronchoscopy, supraglottic airway devices, and emergency front-of-neck airway equipment. Following spinal anesthesia with 3.2 mL of 0.5% hyperbaric bupivacaine and 15 μg of fentanyl, light general anesthesia was induced while preserving spontaneous ventilation. Neuromuscular blockade was avoided, and a size 3 i-gel second-generation supraglottic airway was successfully inserted on the first attempt. Anesthesia was maintained with sevoflurane while spontaneous ventilation was preserved throughout laparoscopic surgery. Pneumoperitoneum was established gradually with intra-abdominal pressure maintained below 12 mmHg. The procedure was completed uneventfully without desaturation, airway obstruction, or significant hemodynamic instability. The i-gel was removed after recovery of consciousness and adequate airway reflexes. Postoperative recovery was uncomplicated, with satisfactory analgesia and no respiratory, neurological, or airway-related complications. This case illustrates that hybrid spinal-general anesthesia using a second-generation supraglottic airway while preserving spontaneous ventilation may be a useful alternative in carefully selected patients with an anticipated difficult airway. Such an approach requires meticulous preoperative airway assessment, experienced anesthetic management, and immediate availability of appropriate rescue airway equipment.

## Introduction

Rheumatoid arthritis (RA) is a chronic multisystem inflammatory disease that can result in progressive joint destruction and significant musculoskeletal deformity. Although peripheral joints are most commonly affected, involvement of the cervical spine, temporomandibular joints, and upper airway structures may have important implications for anesthetic management [1]. Long-standing RA may be associated with atlantoaxial instability, restricted cervical mobility, limited mouth opening, and impaired mandibular movement, all of which can make airway management challenging and increase the potential for complications during airway manipulation [2].

Patients with advanced RA therefore require meticulous preoperative airway assessment and individualized planning. Cervical spine involvement and instability are particularly important considerations because excessive cervical movement during airway instrumentation may increase the potential for neurological complications [3]. In addition, the overall anesthetic management of patients with RA requires consideration of joint deformity, restricted mobility, cervical spine disease, and potential airway difficulties [4].

Awake tracheal intubation, particularly using flexible bronchoscopy, is an established approach when difficult tracheal intubation is anticipated because it permits airway control while spontaneous ventilation is maintained [5]. However, the airway technique should be individualized according to patient anatomy, clinical circumstances, available expertise, and equipment. In selected patients, alternative strategies may be considered when appropriate monitoring, experienced airway personnel, and rescue equipment are immediately available.

Neuraxial anesthesia may provide useful analgesic and anesthetic supplementation during laparoscopic surgery and may reduce the requirement for general anesthetic agents and systemic analgesics [6]. Spinal anesthesia has also been described as a component of anesthetic management for laparoscopic cholecystectomy, demonstrating the feasibility of neuraxial techniques in selected patients undergoing laparoscopic surgery [7].

Difficult airway guidelines emphasize systematic preparation for anticipated airway difficulty, including consideration of alternative airway devices and rescue strategies [8]. Supraglottic airway devices form an important component of modern airway management, and videolaryngoscopy and supraglottic airway techniques have become increasingly incorporated into contemporary airway practice [9]. In patients with RA, the presence of restricted mouth opening, cervical spine abnormalities, and other anatomical predictors may contribute to difficult tracheal intubation and should be considered when selecting an airway strategy [10].

We report the successful perioperative anesthetic management of a patient with severe deforming RA and multiple predictors of an anticipated difficult airway undergoing laparoscopic cholecystectomy. A hybrid spinal-general anesthesia technique was used with a second-generation i-gel supraglottic airway, while neuromuscular blockade was avoided and spontaneous ventilation was maintained throughout the procedure. This case highlights the importance of individualized airway planning and describes the potential role of combining neuraxial anesthesia with carefully controlled general anesthesia in a selected patient with significant anticipated airway difficulty.

## Case presentation

A 54-year-old woman presented for elective laparoscopic cholecystectomy for symptomatic cholelithiasis. She had a history of seropositive RA diagnosed approximately two decades before the current presentation. Despite medical treatment during the early phase of the disease, compliance had been poor over the preceding years, resulting in progressive joint deformities and severe functional disability. She had been wheelchair-bound for approximately 11 years because of advanced RA involving multiple large and small joints.

Her medical history was also significant for depression and anxiety disorder for five years, for which she was receiving olanzapine 2.5 mg once daily, paroxetine controlled release 12.5 mg once daily, and clonazepam 0.5 mg at bedtime. There was no history of ischemic heart disease, diabetes mellitus, hypertension, bronchial asthma, or chronic kidney disease. She denied symptoms suggestive of cricoarytenoid arthritis, including hoarseness, stridor, or dyspnea.

The patient had previously undergone a lower-segment cesarean section and bilateral total knee replacement under spinal anesthesia without perioperative complications.

On physical examination, severe deformities were noted in both hands, including ulnar deviation of the metacarpophalangeal joints, flexion deformities of the wrists and elbows, bilateral knee deformities, and generalized limitation of joint movements (Figure 1).

**Figure 1:**
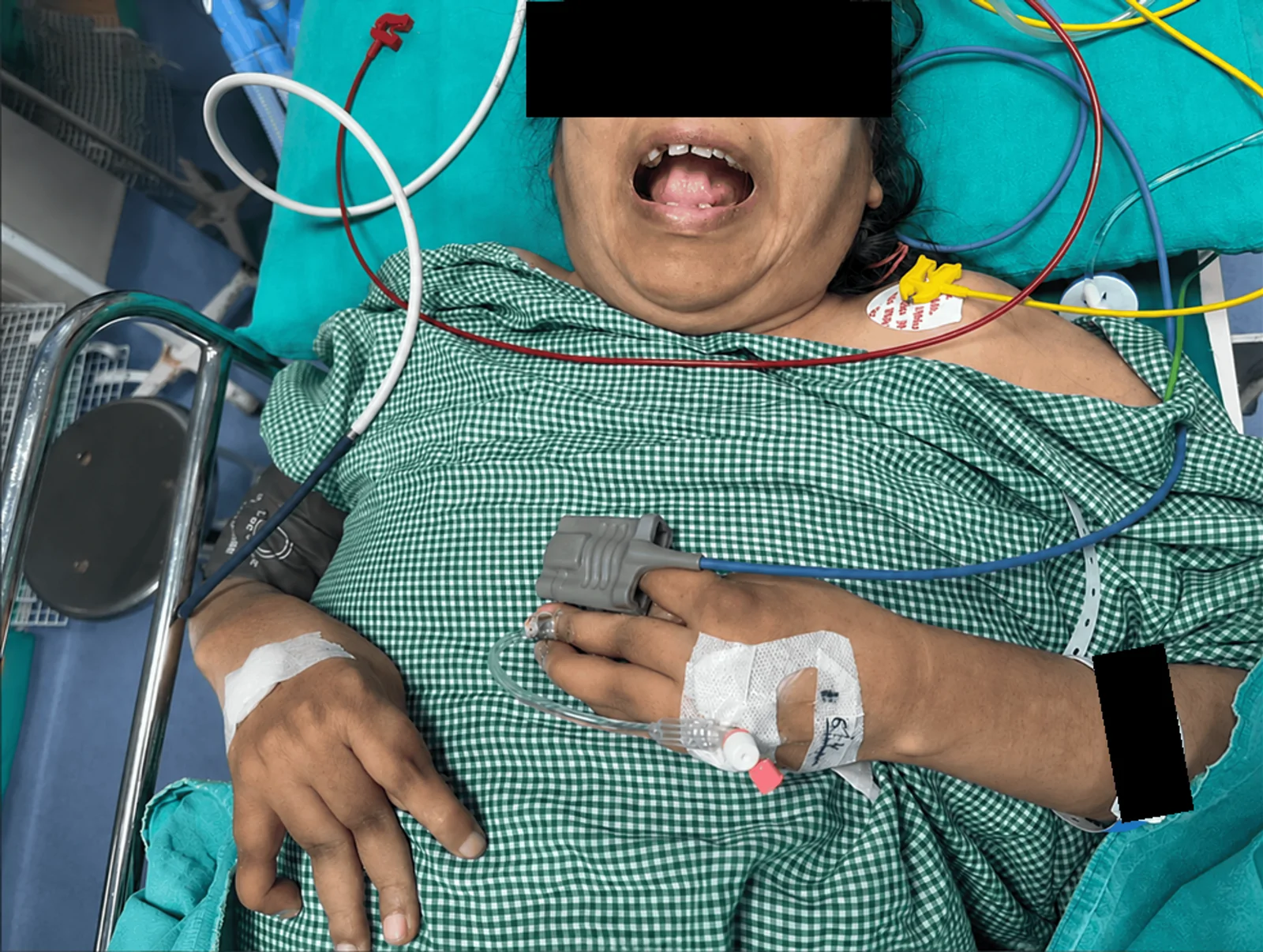
Preoperative clinical photograph demonstrating severe rheumatoid deformities involving both upper limbs, short neck, and limited mouth opening suggestive of an anticipated difficult airway.

Functional capacity was poor, estimated at less than 4 metabolic equivalents. Airway examination revealed a Mallampati grade IV airway, a mouth opening of approximately 1.5 cm, a short neck, a thyromental distance of approximately 5.5 cm, and markedly restricted cervical spine movement with minimal extension [11]. These findings were considered suggestive of anticipated difficult laryngoscopy and tracheal intubation. Baseline oxygen saturation was 94% on room air.

Routine preoperative investigations, including complete blood count, renal and liver function tests, coagulation profile, serum electrolytes, and blood glucose, were within acceptable limits for surgery (Table 1).

**Table 1:** Preoperative laboratory investigations.

Investigation	Result	Reference range
Complete blood count		
Hemoglobin	11.8 g/dL	12-16 g/dL
Total leukocyte count	7,600/mm³	4,000-11,000/mm³
Platelet count	2.4 × 10⁵/mm³	1.5-4.5 × 10⁵/mm³
Renal function tests		
Blood urea nitrogen	15 mg/dL	7-20 mg/dL
Serum creatinine	0.8 mg/dL	0.6-1.2 mg/dL
Liver function tests		
Total bilirubin	0.7 mg/dL	0.2-1.2 mg/dL
Aspartate aminotransferase	24 U/L	10-40 U/L
Alanine aminotransferase	22 U/L	7-56 U/L
Alkaline phosphatase	92 U/L	44-147 U/L
Serum albumin	4.0 g/dL	3.5-5.0 g/dL
Coagulation profile		
Prothrombin time	11.8 seconds	11-13.5 seconds
International normalized ratio	1	0.8-1.2
Activated partial thromboplastin time	29 seconds	25-35 seconds
Serum electrolytes		
Sodium	139 mmol/L	135-145 mmol/L
Potassium	4.1 mmol/L	3.5-5.0 mmol/L
Chloride	103 mmol/L	98-107 mmol/L
Blood glucose		
Random blood glucose	96 mg/dL	70-140 mg/dL

Electrocardiography demonstrated normal sinus rhythm. Transthoracic echocardiography showed preserved left ventricular systolic function with an ejection fraction of 60%. Lower-limb venous Doppler examination showed no evidence of deep vein thrombosis.

Considering the anticipated difficult airway, a detailed perioperative airway management plan was formulated. A comprehensive difficult airway cart containing a videolaryngoscope, fiberoptic bronchoscope, various supraglottic airway devices, bougies, stylets, and emergency front-of-neck airway equipment was kept immediately available. The primary objective was to minimize airway manipulation while maintaining spontaneous ventilation. The patient was counseled regarding the proposed anesthetic technique and the possibility of requiring advanced airway management, and written informed consent was obtained.

Anesthetic management

After confirmation of adequate fasting, the patient was transferred to the operating room. Standard American Society of Anesthesiologists monitoring, including continuous electrocardiography, noninvasive blood pressure, pulse oximetry, capnography, and temperature monitoring, was instituted. An 18-G IV cannula was secured, and lactated Ringer’s solution was commenced.

Given the anticipated difficult airway, the difficult airway cart was positioned and immediately available before induction. The surgical team was informed of the airway concerns and the planned airway strategy.

Under strict aseptic precautions, spinal anesthesia was administered in the sitting position at the L3-L4 intervertebral space using a 25-G Quincke spinal needle. Following confirmation of free flow of cerebrospinal fluid, 3.2 mL of 0.5% hyperbaric bupivacaine with 15 μg of fentanyl was administered intrathecally. The patient was then positioned supine, and an adequate sensory block up to the T4 dermatome was achieved.

After confirmation of a satisfactory neuraxial block, light general anesthesia was induced with IV propofol administered in titrated doses while preserving spontaneous ventilation. Neuromuscular blockade was intentionally avoided because loss of spontaneous ventilation in a patient with an anticipated difficult airway could increase the risk of inability to ventilate if airway management proved difficult.

A size 3 i-gel supraglottic airway (Intersurgical Ltd., Wokingham, UK) was inserted smoothly on the first attempt without difficulty. Correct placement was confirmed by bilateral chest expansion, auscultation, square-wave capnography, and adequate tidal volume. Anesthesia was maintained with an oxygen-air mixture and sevoflurane, while spontaneous breathing was maintained with pressure support when required.

Pneumoperitoneum was established gradually, and intra-abdominal pressure was maintained below 12 mmHg throughout the laparoscopic procedure to minimize respiratory compromise. The patient remained hemodynamically stable without episodes of desaturation, airway obstruction, or significant fluctuations in heart rate or blood pressure. End-tidal carbon dioxide remained within acceptable limits throughout the procedure.

Laparoscopic cholecystectomy was completed successfully without conversion to open surgery. At the completion of the procedure, anesthetic agents were discontinued, and the patient regained consciousness with intact spontaneous ventilation. The i-gel was removed after she responded appropriately to verbal commands and demonstrated adequate airway reflexes. She was transferred to the post-anesthesia care unit for routine monitoring.

The postoperative period was uneventful. Analgesia was adequately maintained without excessive opioid requirements, attributed to the residual neuraxial analgesic effect. No respiratory complications, neurological deficits, airway-related events, postoperative nausea, or vomiting were observed. The patient fulfilled standard recovery criteria and was subsequently discharged from the recovery area.

## Discussion

Anesthetic management of patients with advanced RA can be challenging because of potential involvement of the cervical spine, temporomandibular joints, cricoarytenoid joints, and peripheral joints. These manifestations may significantly affect airway management and positioning during anesthesia [2,4]. Importantly, airway abnormalities and difficult intubation may occur in patients with RA, highlighting the need for detailed preoperative assessment and individualized airway planning [2,4,10].

In the present case, the patient had several features suggestive of an anticipated difficult airway, including a Mallampati grade IV airway, markedly restricted mouth opening, a short neck, and severe limitation of cervical spine movement. These findings raised concerns regarding both airway instrumentation and the potential consequences of excessive cervical manipulation. Cervical spine abnormalities in patients with RA may increase the potential risk associated with excessive neck movement during airway management [2-4]. Although our patient did not have clinical features suggestive of cricoarytenoid involvement, such as hoarseness, stridor, or dyspnea, this possibility should be considered during preoperative assessment of patients with advanced RA [4].

Airway management considerations

The principal objective of airway management in an anticipated difficult airway is maintenance of oxygenation while minimizing potentially harmful airway manipulation. The American Society of Anesthesiologists Difficult Airway Guidelines emphasize individualized airway assessment and preparation according to the anticipated difficulty with ventilation, laryngoscopy, intubation, and rescue oxygenation [1]. Similarly, difficult airway guidelines emphasize maintaining oxygenation and having an appropriate rescue strategy available when airway difficulty is anticipated [8].

Awake tracheal intubation using flexible bronchoscopy remains an established approach for many patients with an anticipated difficult airway because it permits airway control while spontaneous ventilation is preserved [5]. However, the choice of airway technique should be individualized according to patient characteristics, clinical circumstances, available equipment, operator expertise, and patient cooperation. In the present case, the anesthetic plan was designed around minimizing airway manipulation and maintaining spontaneous ventilation, with a comprehensive difficult airway cart and emergency front-of-neck airway equipment immediately available.

Preservation of spontaneous ventilation and avoidance of neuromuscular blockade

Preservation of spontaneous ventilation was a central component of our anesthetic strategy. In a patient with significant predictors of a difficult airway, administration of a neuromuscular blocking agent before successful establishment of a reliable airway may result in loss of spontaneous respiratory effort and potentially make ventilation more difficult if the selected airway device cannot be inserted or does not provide adequate ventilation.

For this reason, neuromuscular blockade was intentionally avoided in our patient. Following establishment of an adequate spinal block, light general anesthesia was induced using titrated propofol while spontaneous ventilation was maintained. This approach allowed the patient to continue breathing spontaneously during airway establishment and subsequent maintenance of anesthesia. Importantly, avoidance of neuromuscular blockade should not be interpreted as eliminating the need for a complete difficult airway strategy. Alternative airway devices, fiberoptic equipment, and emergency airway equipment remained immediately available throughout the procedure [1,8].

Role of the second-generation supraglottic airway

Supraglottic airway devices are established components of airway management and may provide an alternative means of ventilation in appropriately selected patients. Difficult airway guidelines recognize supraglottic airway devices as important components of airway rescue and emphasize preparation for failure and availability of alternative airway devices [8]. Videolaryngoscopy has also become an established component of contemporary difficult airway practice [9], although it represents a different airway management modality from the supraglottic airway used in the present case.

In our patient, a size 3 i-gel was successfully inserted on the first attempt after induction of light general anesthesia, and effective ventilation was confirmed clinically and by capnography. The device remained effective throughout laparoscopic surgery, including during pneumoperitoneum. Its use also avoided direct laryngoscopy and tracheal instrumentation in a patient with markedly restricted mouth opening and cervical spine movement.

Nevertheless, a supraglottic airway should not automatically be considered an alternative to tracheal intubation in every patient with an anticipated difficult airway. Patient selection is important, particularly in laparoscopic surgery, where pneumoperitoneum and changes in intra-abdominal pressure can influence respiratory mechanics. In addition, a supraglottic airway does not provide the same degree of airway protection from aspiration as a cuffed endotracheal tube. In the present case, its use was based on the individual clinical circumstances, the successful establishment of an adequate neuraxial block, preservation of spontaneous ventilation, and immediate availability of alternative airway and rescue equipment [8].

Role of spinal anesthesia as an adjunct to general anesthesia

The use of spinal anesthesia was an important component of the hybrid technique. Neuraxial anesthesia can provide substantial afferent analgesia and may reduce the requirement for systemic and inhalational anesthetic agents during surgery [6,7]. In laparoscopic procedures, spinal anesthesia has been described as an adjunct to general anesthesia in selected patients, potentially allowing a lighter plane of general anesthesia while providing effective perioperative analgesia [6,7].

In this patient, spinal anesthesia with hyperbaric bupivacaine and fentanyl produced a sensory block up to the T4 dermatome before induction of general anesthesia. This provided an additional analgesic component during laparoscopic cholecystectomy and contributed to satisfactory postoperative analgesia. The combination allowed the general anesthetic to be maintained with sevoflurane while spontaneous ventilation was preserved and neuromuscular blockade was avoided.

The hybrid approach should therefore be viewed as a combination of complementary techniques rather than as a replacement for established difficult-airway strategies. The neuraxial component addressed surgical nociception and postoperative analgesia, while the light general anesthetic and supraglottic airway provided the conditions required for laparoscopic surgery without tracheal intubation or neuromuscular blockade.

Perioperative airway planning in laparoscopic surgery

Laparoscopic surgery presents additional considerations in patients with an anticipated difficult airway because pneumoperitoneum can alter respiratory mechanics and increase ventilatory requirements. In our patient, pneumoperitoneum was established gradually, and intra-abdominal pressure was maintained below 12 mmHg. Spontaneous ventilation was preserved throughout the procedure, with pressure support used when required. End-tidal carbon dioxide remained within acceptable limits, and there were no episodes of desaturation or airway obstruction.

The successful intraoperative course does not imply that this approach is appropriate for all patients with an anticipated difficult airway undergoing laparoscopic surgery. Rather, it demonstrates the importance of integrating the airway assessment, surgical requirements, neuraxial block, ventilation strategy, and availability of rescue equipment into a single individualized anesthetic plan [6,8].

Clinical significance of the combined technique

Several airway management techniques have been described for patients with RA, including awake fiberoptic intubation and other advanced airway approaches [2,4,5]. Difficult intubation has also been specifically described in patients with RA, supporting the importance of careful airway assessment and individualized airway planning in this population [10]. Videolaryngoscopy represents another established airway management modality [9]. Similarly, spinal anesthesia and supraglottic airway devices are established techniques in selected laparoscopic procedures [6-8].

However, reports describing the deliberate combination of spinal anesthesia, light general anesthesia, a second-generation supraglottic airway, preservation of spontaneous ventilation, and avoidance of neuromuscular blockade in a patient with severe deforming RA and multiple predictors of difficult airway are limited.

The clinical significance of this case therefore lies in the integration of these established techniques into an individualized perioperative strategy. Rather than relying on a single airway or anesthetic technique, the approach was based on minimizing airway manipulation, maintaining spontaneous ventilation, providing adequate surgical anesthesia and analgesia, and ensuring immediate access to rescue airway equipment.

Limitations

This report describes the successful management of a single patient and therefore cannot establish the safety, superiority, or generalizability of this technique. Awake tracheal intubation remains an established approach for many patients with an anticipated difficult airway, and the suitability of a supraglottic airway depends on individual patient and surgical factors [5,8]. Furthermore, successful ventilation through an i-gel in this patient does not establish that the same strategy would be appropriate in patients with different airway anatomy, aspiration risk, respiratory pathology, or more extensive laparoscopic procedures.

The technique should therefore be considered only in carefully selected patients and performed by clinicians experienced in difficult airway management, with continuous monitoring and immediate availability of alternative airway devices and emergency rescue techniques [1,8].

## Conclusions

Patients with severe RA and an anticipated difficult airway require meticulous preoperative assessment and individualized anesthetic planning. In the present case, hybrid spinal-general anesthesia using a second-generation i-gel supraglottic airway, while avoiding neuromuscular blockade and preserving spontaneous ventilation, provided satisfactory surgical conditions and an uncomplicated perioperative course. The combination of neuraxial anesthesia with light general anesthesia may be a useful strategy in carefully selected patients when minimizing airway manipulation is desirable. However, this approach should not be considered a substitute for established difficult-airway techniques in all patients and requires experienced airway management, appropriate patient selection, continuous monitoring, and immediate availability of alternative and rescue airway equipment.
